# Trust over repeated interactions: Majority group members generalize more from interactions with non-coethnic partners

**DOI:** 10.1371/journal.pone.0341143

**Published:** 2026-03-10

**Authors:** Siyeona Chang, Maria Abascal, Delia Baldassarri

**Affiliations:** 1 Independent Researcher; 2 Department of Sociology, New York University, New York, New York, United States of America; Yeditepe University, TÜRKIYE

## Abstract

People increasingly live in complex, heterogeneous communities characterized by differentiation, where groups may lack the shared history and/or close ties that can nurture trust in more traditional communities. How does trust generalize from an interaction with one stranger––either trustworthy or untrustworthy––to subsequent interactions with other strangers? Does trust generalize more from or toward interactions with outgroup members than ingroup members? And, does an interaction with one stranger affect someone’s willingness to interact with strangers from the same group moving forward? This study examines these questions using a repeated trust game in which 1,255 US White adults were paired with a White or Latino partner who behaved in a trustworthy or untrustworthy way. Results reveal that participants paired with an untrustworthy outgroup member in the first round were less likely to choose an outgroup partner for the second round. This effect is pronounced among participants who likely were uncertain initially about their partner’s behavior. These participants also gave less in the second round when their first-round partner behaved in an untrustworthy way. Our findings highlight the need to treat a willingness to interact with outgroup members as an outcome of intergroup contact.

## Introduction

How do people learn whom to trust? In close-knit, homogeneous communities, multiple mechanisms promote trust, including sanctioning of non-cooperative behavior, reciprocity, and reputation/signaling. All of these rely on some degree of familiarity between group members [1–8]. For instance, members of close-knit communities have incentives to be trustworthy because possibilities for repeated interactions create opportunities for direct reciprocity as well as indirect reciprocity, which may hinge on reputation. However, people increasingly live in complex, heterogeneous communities characterized by differentiation and individualization, where groups may lack the shared history and/or close ties that can nurture trust in more traditional communities [9,10]. In complex, heterogeneous contexts, how do people learn to trust strangers, including those from different racial or ethnic backgrounds?

The diversification of modern societies highlights the need for research on how trust extends beyond group boundaries. A literature on contact generalization offers insights into how individuals may develop trust in contexts where they lack direct prior knowledge or reputational information about others. Primary transfer effects of intergroup contact posit that individuals form expectations about strangers by generalizing from their experiences with an individual to the larger group to which that individual belongs [11]. In other words, when dealing with a stranger in a situation that requires trust, individuals may infer how trustworthy that stranger is based on the behavior of similar others with whom they have interacted in the past. Similarity may refer to *perceptual* similarity between known individuals and unfamiliar strangers who share similar physical attributes [12]. Similarity may also refer to *situational* similarity, as is the case in research that alludes to past interactions to explain why people in market-integrated societies are more trusting of strangers [13–15]. (People in less close-knit contexts and market-based societies have more opportunities and incentives to interact with strangers (see also [16]), and these interactions inform subsequent encounters with other strangers.)

Research on intergroup contact has focused on how members of dominant and majority groups (hereafter “majority group” for simplicity) generalize from interactions with outgroup members to other outgroup members. Recent studies examine the impact of being assigned to a roommate of a different race in the military [17], in dorms [18,19], or to classmates from different socioeconomic backgrounds in school [20]. They find that contact leads to greater support for policies aimed at reducing inequality, and to a greater likelihood that individuals exposed to outgroup members will choose another partner from the outgroup in the future, especially when the outgroup member has high aptitude ([19]; but see [21–23] for examples where changes are confined to behaviors toward the individuals directly involved in the interaction). It remains to be seen, however, whether more casual encounters generalize to other outgroup members, and whether majority group members generalize in the same way from an interaction with an outgroup member versus an ingroup member.

There are reasons to expect that generalization may be stronger following interactions with outgroup members, at least for majority group members. First, people may have fewer opportunities to interact with outgroup members than ingroup members. In the United States, for example, most metropolitan statistical areas are still more than 50% White [24], which suggests that the average White American has more encounters with other Whites than with Non-White Americans. Hence, a single encounter with outgroup members can carry more information, reducing uncertainty about how outgroup strangers would behave in future interactions to a greater extent. (A one-off interaction with an untrustworthy ingroup member will not significantly change how someone with a long history of interacting with ingroup members views or treats ingroup members subsequently.) Generalization following interactions with outgroup members may also be stronger if people view outgroup members as “more like each other,” and attribute the behavior of one group member to dispositions of the group generally (see [25] on “outgroup homogeneity bias”). Relatedly, past studies show that people evaluate interaction with ingroup members more favorably than interactions with outgroup members, even in minimal group settings ([26–28] for classic experiments; [29] for a review of differential attribution), which suggests that people may extrapolate differently from experiences with outgroup versus ingroup members. In spite of several such indications that the generalization process may differ depending on the social identity of alter, to our knowledge, no study has offered a direct empirical test of these conjectures.

In this paper, we consider the trusting behavior of White Americans towards other White Americans (ingroup members/coethnics) and Latino Americans (outgroup members/non-coethnics). Using a repeated trust game [30], we test whether the nature of an initial interaction with one person (who is either trustworthy or untrustworthy) affects how participants behave in a subsequent interaction with a different person, and whether these effects differ depending on whether the second-round interaction partner is a coethnic or a non-coethnic.

If generalization varies depending on the group membership of alters, this could have significant consequences. For one, it would imply that the actions of an individual would be seen as more representative of their ethnic group if they belong to a minority, compared to those who belong to the majority [31,32]. Individuals who are part of a minority group would find that their behaviors could have disproportionate effects on others within their group, and that they themselves are more likely to be judged by the behavior of their coethnics, compared to those who belong to the majority group. While our study does not directly measure the tokenism burden on minorities, by comparing how majority group members generalize after interacting with coethnics and non-coethnics, we explore whether and to what extent tokenism burden may be rooted in actual generalization patterns.

This study contributes to existing research in four key ways: First, using a trust game that involves a one-shot, minimal interaction, our study examines primary transfer effects in a condition that mimics the kind of interactions that people are likely to have in complex, diverse societies. Our approach complements existing research which focuses on contact involving intimate, long-term interactions, such as being roommates, whether in the military [17] or in a college dorm [18,19]. Such “rarefied” settings may be the exception rather than the norm ([21], p. 1809).

Second, our design provides a first known direct comparison of how members of the majority group generalize from their experiences with ingroup members to other ingroup members, as opposed to outgroup members to other outgroup members. Understanding whether and how majority group members generalize differently from experiences with outgroup and ingroup members can shed light on the unique challenges that minorities face in contexts in which they are underrepresented.

Third, in contrast to research on intergroup contact, which focuses on whether and how contact affects attitudes and behavior towards outgroup members––under the assumption that contact will subsequently take place––our study also examines whether and how contact affects the decision to engage in a subsequent interaction with an outgroup versus ingroup member. The existence of opportunities for contact does not necessarily translate into actual contact or improved intergroup attitudes/relations [33], and yet, as highlighted by Baldassarri et al. [34], the role of selection into intergroup interaction has largely been overlooked. Through our examination of the selection process, our findings complement previous research, providing a more comprehensive understanding of the consequences of intergroup contact.

Fourth, and finally, we employ behavioral measures of contact outcome, heeding the call to use behavioral measures in a field where self-reported attitudinal measures are common [35].

Specifically, we test the following hypotheses about the effect of first-round interaction on second-round partner selection:

- H1. Participants who are paired with a trustworthy (versus untrustworthy) partner in the first round will be more likely to select a second-round partner of the same race/ethnicity.
- H2. Participants will be more likely to select a White (coethnic) versus Latino (non-coethnic) partner for the second round.

In addition to assessing how the first-round interaction influences subsequent partner selection, we also examine people’s behavior when they play with an assigned partner. In this setup, the amount individuals decide to send to their partner (H3) is interpreted as a measure of trust. We also test whether the tendency to generalize from an initial interaction to a subsequent interaction is stronger when participants in both interactions are of the same ethnicity (H4). Finally, we test whether the tendency to generalize from an initial interaction to a subsequent interaction is stronger if both interactions are with an outgroup (Latino) partner (H5).

In summary:

- H3. Participants who are paired with a trustworthy partner in the first round will send more money to their partner in the second round than will participants who are paired with an untrustworthy partner in the first round.
- H4. The effect of being paired with a trustworthy versus untrustworthy first-round partner on second-round contributions will be stronger among participants whose partners are both White or both Latino than among participants with one White partner and one Latino partner.
- H5a. The effect of being paired with a trustworthy versus untrustworthy first-round partner on second-round contributions will be stronger among participants who are paired with a Latino partner in the second round than among participants who are paired with a White partner in the second round.
- H5b. The effect of being paired with a trustworthy versus untrustworthy first-round partner on second-round contributions will be stronger among participants who are paired with a Latino partner in the first round than among participants who are paired with a White partner in the first round.

To anticipate our results, we find that the behavior of an outgroup partner in the first round (trustworthy versus untrustworthy) affects behavior in a second round. Specifically, White participants who are treated in an untrustworthy way in the first round by a Latino partner are less likely to choose another Latino partner for the second round than those treated in a trustworthy way by a Latino partner. This effect is pronounced among participants who initially gave some but not all of their endowments, a proxy for initial uncertainty. By contrast, the first-round behavior by a White partner does not affect contributions in the second round. Further, first-round partner behavior does not affect contributions in the second round when second-round partners are assigned by the experimenter. However, subgroup analyses suggest that those who gave some but not all of their endowments give less in the second round when their first-round partner behaved in an untrustworthy way. In this regard, our study suggests that the partner selection process constitutes a component of intergroup contact that is particularly sensitive to generalization, as well as to persistent outgroup bias. At the same time, heterogeneity revealed by subgroup analyses highlights the merits of understanding how baseline individual orientations toward uncertainty may moderate the effects of contact.

### Data and methods

We fielded an experiment involving repeated trust games with 1,255 White-identifying US resident adults, recruited via Amazon Mechanical Turk (MTurk) in May 2022. All participants played two rounds of a trust game. The goal was to understand how behavior in the second round (contributions and partner selection) was affected by a trustworthy or untrustworthy interaction in the first round with a coethnic (White) or non-coethnic (Latino) partner. Our design, questions, and hypotheses were registered online prior to the collection of outcome data (https://osf.io/8kt9a/overview). The experiment was approved by NYU’s Institutional Review Board: Trust Over Repeated Interactions (IRB-FY2021–4943) and Columbia University’s Institutional Review Board: Trust Over Repeated Interactions (AAAU0173(M01Y01)). Participants provided informed, written consent at the beginning of the survey. All participants were 18 years or older.

### Experimental design

**Fig 1:**
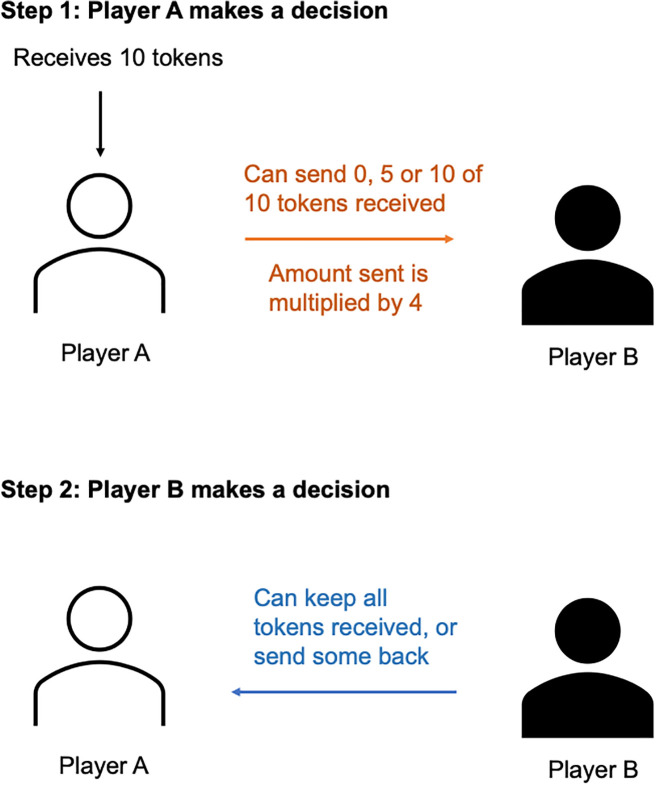
Setup of the trust game.

The trust game involves two participants, a first mover and a second mover, or Player A and Player B. In our design, Player A received $0.50 as 10 tokens and decided how many tokens, if any, to send to Player B. Player A could send 0, 5 or 10 tokens. The amount Player A sent was multiplied by four, then Player B decided how much of the total received to return to Player A (Fig 1). Player B had two choices: To keep the full amount received and return nothing, or to keep some of the amount received and return the rest.

Player A’s behavior represents her trust toward Player B, and Player B’s behavior represents her trustworthiness toward Player A. Research has validated Player A’s behavior with self-reported trusting behavior in the real world, including lending money and possessions and leaving doors unlocked [36]. Research has also validated Player B’s behavior (trustworthiness) with loan repayment [37] and donating money [38] in the real world. More recently [39] validates trust game behavior with generalized self-reported trust ([40] for a review of trust games). While there are alternative interpretations of Player A’s behavior in the trust game, such as altruism—as opposed to trust—the altruism account has been empirically tested and found no support [41]. We therefore follow the interpretation of Player A’s behavior as representing trust, as is routinely done in studies using trust games.

In our experiment, we follow Ermisch et al. [42] by providing participants with discrete choices, because they better capture the binary nature of risk in interactions: people can either trust others—for example by lending them their car, hiring them, etc.—or not. We also provide discrete choices because results from numerous past behavioral games reveal that contributions tend to form a trimodal distribution with modes at the minimum, maximum, and middle values [43].

The focus of the study was on the behavior of Player A, the first mover, and thus the majority (N = 1,215) of the participants were assigned to the role of Player A, although a small minority of participants (N = 40) were assigned to the role of Player B. In the first round, participants were paired with a coethnic (White) or non-coethnic (Latino) partner in the role of Player B. Player B either behaved in a trustworthy way (keeping 18 tokens and sending back 22 out of the 40 tokens received, or keeping 8 tokens and sending back 12 out of the 20 tokens received) or an untrustworthy way (keeping all of the money received). The ethnicity of Player B as well as Player B’s behavior were randomly assigned. Player B was programmed as a bot because, with the majority of MTurk workers being White, and with Latino workers making up a relatively small share in comparison, it would not have been possible to recruit a large enough Latino sample in a way that allowed for real-time matching within budgetary constraints. A majority of MTurk workers identify as White (https://www.cloudresearch.com/resources/blog/who-uses-amazon-mturk-2020-demographics/), as do a majority of US Americans. Relying on chance to pair White Player As with a sufficient number of Latino Player Bs would have meant collecting a much larger number of White Player Bs than would have been required. Moreover, by programming Player B as a bot, we were able to ensure a comparable number of observations in each experimental condition (trustworthy versus untrustworthy).

Lastly, a bot allowed us to randomly assign other demographic characteristics (age, income) to Player B, ensuring that the effect of ethnicity was not confounded with correlated traits [44]. Specifically, among the participants, 33.71% were assigned to the 25–29 age range, 33.15% to the 30–34 range, and another 33.15% to the 35–39 range. In terms of income, 33.94% were assigned to the $40,000–49,999 range, another 33.94% to the $50,000–59,999 range, and 32.11% to the $60,000–69,999 range. Finally, by programming Player B as a bot, we were able to pair participants with partners of the same gender, so as to focus on the effect of ethnicity. This decision was informed by previous research carried out by one of the authors in which men tend to be significantly more generous in trust games when paired with female partners (more information available upon request).

**Fig 2:**
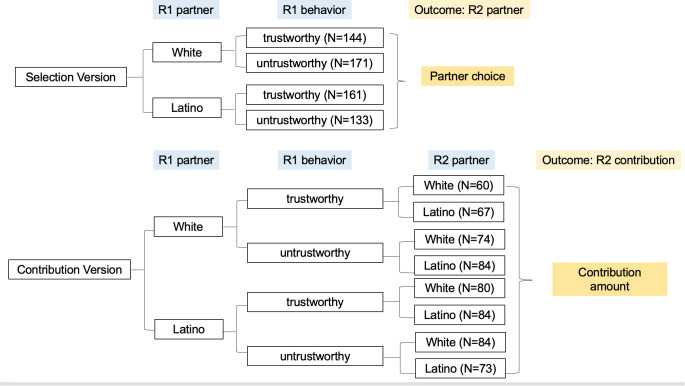
Experimental conditions and the number of observations.

For the second round, participants were also randomly assigned to one of two versions of the trust game. In the selection version, participants––who had been randomly assigned to a partner for the first round––chose their partner for the second round. Specifically, they could choose from a White partner and a Latino partner. Their choice was the outcome of interest. In the contribution version, the second round was identical to the first; participants were randomly assigned to a partner in both rounds and the outcome of interest was contribution to the second-round partner, whose ethnicity was randomly assigned as before. In summary, participants in the role of Player A could be assigned to any one of the 12 experimental conditions: 4 conditions in the selection version, depending on the identity and behavior of the first-round partner; and 8 conditions in the contribution version, depending on the identity of the first-round partner, the behavior of the first-round partner, and the identity of the second-round partner. Fig 2 illustrates these conditions and the distribution of participants across them.

After completing both rounds of the trust game, participants took part in a face recognition task. In this task, they first viewed pictures of 24 faces (12 White and 12 Latino) from the Chicago Face Database [45]. (The faces selected were characterized by low rates of misclassification in the norming data provided with the CFD.) Then, they answered a series of questions about themselves (e.g., educational attainment, economic satisfaction, nativity, vote choice). Finally, returning to the face recognition task, they viewed pictures of 36 faces and answered whether they had seen each face earlier. The face recognition task was intended to capture participants’ relative familiarity with individuals of White and Latino background, to test whether familiarity moderated the effects of the ethnicity and trustworthiness of the alter on subsequent behavior. (See S2 Text for more on this task and the description of the associated sensitivity measure.) A rich literature on the effects of interpersonal contact [35,46] and parasocial contact (e.g., [47]) anticipates that familiarity may temper bias toward outgroups.

### Treatment of those who did not send anything

We were interested in observing how Player A generalizes from Player B’s behavior in the first round to a subsequent round. However, Player As who chose not to send any tokens to Player B could not directly observe Player B behaving in a trustworthy or untrustworthy way. (As in the real world, trustworthiness was revealed only following the decision to trust.) However, in order to collect meaningful behavior for those who gave 0 tokens, we introduced a small modification to the trust game: before finding out whether and how much Player A had actually sent, Player B was asked to decide whether they would send back any tokens in each scenario (i.e., 5 tokens and 10 tokens). When Player As were shown the outcome of the game, they received information both about what Player B would have done in each scenario alongside the realized outcome, determined by what they had actually sent to Player B.

In this way, Player As who gave 5 or 10 tokens had both knowledge of what Player B *would have done* in each scenario, as well as first-hand experience of Player B’s actual behavior. Not having sent any tokens that could be kept or returned, Player As who sent 0 tokens were not “treated” in the same way as those who sent 5 or 10. But, by providing information about what Player B would have done, we test whether a treatment that is hypothetical in nature could itself generalize onto subsequent interactions.

### Participants

The analyses that follow are based on 1,255 participants. Participants were eligible to participate if they identified as White, resided in the United States, had a higher than a 95% acceptance rate on previous work submitted on the platform, and correctly answered basic comprehension check questions. Prospective participants were asked to enter basic demographic information at the beginning of the survey to determine eligibility. Those who passed the screener were given instructions about the activities and took comprehension check questions to confirm their understanding. Participants who filled out the screening survey but were not eligible (non-White) were paid $0.05 in compensation for their time. Participants who were eligible but incorrectly answered the comprehension check questions twice were disqualified from participating in the activities, and paid $0.10 in compensation for their time. Participants were paid $3.00 for completing the experiment, and up to an additional $2.00 based on the outcome of the games. The experiment took 17.5 minutes on average.

MTurk is a widely used platform for experimental research. The average MTurk user tends to be more educated and more likely to lean Democrat than the average US adult [48]. While concerns about the generalizability of findings from the convenience sample have been raised, recent work has shown that results based on MTurk samples closely mirror results based on representative, random samples [49]. Moreover, although MTurk samples differ from the broader US population in certain ways, they include sufficient variation to allow for subgroup analyses where appropriate. In our sample, 73.3% of participants were between the ages of 18–39 years old, 60% earned between $40,000 and $79,999 per year, and 62.5% identified as Democrats (S1 Table). Moreover, 65.5% of the sample identified as Male, consistent with other recent studies which have sampled from MTurk [48]. We conducted additional analyses to ensure that the gender imbalance did not bias our findings. We found that while men tend to contribute more, there were no significant interactions between gender and treatment conditions. In addition, we found all of the treatment groups to be balanced along participant demographics (e.g., age, gender), as expected.

## Results

### The effect of a trustworthy or untrustworthy interaction on partner selection

We hypothesized that the nature of first-round interaction would impact the decision about whom to play with in the second round. Specifically, we predicted that participants who were paired with a trustworthy (versus untrustworthy) partner in the first round would be more likely to select a second-round partner of the same ethnicity as their first-round partner (H1). It is worth noting at the outset that participants demonstrated a preference for White partners, regardless of the treatment condition, consistent with H2. Even among those who had a trustworthy interaction with a Latino partner in the first round, the majority chose to play with a White partner in the second round. Moreover, among White participants paired with an untrustworthy White partner in the first round, the majority of (57.3%) still chose a White partner for the second round. (Supplementary analyses reveal that the odds that respondents chose a Latino partner in round 2 were 34% lower for Republicans than Democrats (p = .066), controlling for experimental conditions. Partisanship was the only individual- or ZIP-code-level characteristic to significantly, linearly predict partner choice.) The preference for a co-ethnic partner among White participants independent of the effect of first-round interaction suggests that unlike much of the contact literature which measures the effects of interactions on intergroup relations conditional on contact taking place, when left up to people, contact itself cannot be taken for granted.

**Fig 3:**
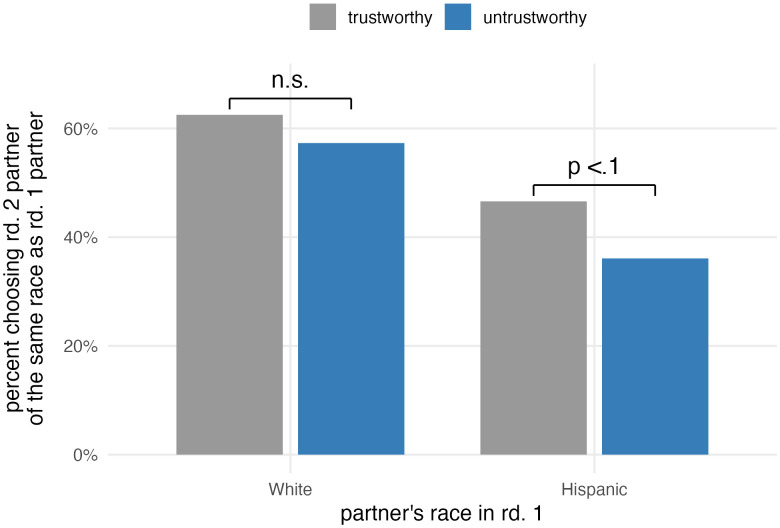
Effect of treatment on partner choice in round 2.

In terms of the effect of the first-round interaction, as Fig 3 shows, we find that those who had a positive/trustworthy interaction in round 1 were more likely to choose to play with another partner of the same ethnicity as in round 1, but that the magnitude of the impact differs depending on the ethnicity of the round 1 partner.

Among participants who were paired with a Latino partner in the first round, 46.6% of those who had a trustworthy interaction chose a Latino partner in round 2, whereas only 36.1% did among those who had an untrustworthy interaction. In other words, participants paired with a Latino partner who acted in an untrustworthy way were less likely to choose to interact with another Latino partner. On the other hand, among participants who were paired with a White partner in the first round, 62.5% of those who had a trustworthy interaction chose a White partner, whereas 57.3% did among those who had an untrustworthy interaction (See S3 Table for complete percentages). This difference is in the anticipated direction, but it is not statistically significant (p = 0.106) (Table 1).

**Table 1:** Regression estimates: Selecting an R2 partner of the same race as R1, as a function of partner ethnicity and trustworthiness in R1.

Predictor	R1 partner = White	R1 partner = Latino
Untrustworthy behavior	−0.419 (0.258)	−0.450’ (0.262)
N	315	294

*Note:* ‘p < 0.1; *p < 0.05; **p < 0.01; ***p < 0.001

In sum, the first-round partner’s behavior (trustworthy versus untrustworthy) has a bigger impact on partner selection when the first-round player is non-coethnic (Latino). This finding offers evidence in support of the hypothesis that the way contact generalizes from an individual to a broader set of strangers differs depending on whether the stranger is a member of the ingroup versus outgroup. Our study design does not distinguish between several possible explanations for this finding; it is possible, for instance, that information about a relatively unfamiliar group is more impactful than information about a familiar group. (Given that our sample consists of White Americans, we assume they have more prior experience interacting with other Whites than with Latinos, on average.) However, the results indicate that members of the majority group not only generalize from a single experience with a member of the outgroup to other members of that group, but that they do so when they would not make the same generalization if the same interaction were to involve an ingroup member. Sensitivity on the face recognition task does not moderate the effect of the first-round partner’s behavior on selection among participants paired with Latino partners in the first round. This would have further supported our interpretation of this result as rooted in familiarity. However, the task yielded limited variation in recognition rates between Whites and Latinos, suggesting our face recognition task was a noisy proxy for familiarity.

To examine the role of familiarity further, we linked respondents to ZIP-code area characteristics from the 2022 American Community Survey. The percentage of Hispanic residents in a respondent’s ZIP code did not significantly moderate the effect of untrustworthy behavior on second-round partner selection (or contributions), controlling for other ZIP code area differences. Notably, the contact literature also finds weaker effects for real-world contact than experimentally induced contact in the lab [35].

### Partner choice—conditional average treatment effect

We also estimated the conditional average treatment effects by initial contribution amount, which we interpret as an indicator for the baseline propensity to trust. The following results are based on post-hoc analyses that were not pre-registered. Recall that Player As were able to send nothing (0 tokens), to send half of their endowment (5 tokens), or to send all of their endowment (10 tokens).

These discrete options, however, can also map onto an initial disposition towards uncertainty. For instance, those who give 0 tokens in the first round are likely more risk-averse and less trusting of others, and therefore less likely to change their behavior based on a single interaction. Those who sent 10 tokens, on the other hand, may be willing to take risks and/or to think others are very likely to send money back. On the other hand, the decision to give 5 tokens, in simultaneously ensuring a minimum compensation for oneself while risking the rest of the endowment for a larger potential return, may indicate that a participant feels more uncertain about the outcome than those participants who give nothing or all. Taken together, this reasoning suggests that the conditional average treatment effects may be stronger among the subgroup of participants that is “most uncertain” in their expectations about partners.

We use the notion of uncertainty instead of risk-taking, although the latter is more common in the literature. As De Groot & Thurik [50] note, *risk* refers to decision-making in situations where the decision outcomes and their probabilities of occurrences are known to the decision-maker, whereas the situation in the trust game involves *uncertainty*, where the probability that the trustee will send back some of the money received is unknown to the decision-maker. Moreover, within the literature on the relationship between trusting behavior and risk attitudes, empirical evidence remains mixed. Numerous studies, including seminal work such as Eckel & Wilson [51] find that risk attitudes do not predict the amount trustors send in a trust game, whereas recent studies suggest that the risk averse tend to trust less [52,53]. In our study, a survey measure of risk was not correlated with the amount participants shared. Also, in our study, it was those who opted for the middle option in the first round that were most likely to change their behavior in response to the first round. In this regard, the pattern in our data does not correspond to existing theoretical conjectures about the (linear) relationship between risk-taking and trust behavior.

Another reason for the subgroup analysis is that participants who gave 0 tokens experienced trustworthiness and untrustworthiness differently than those who gave 5 or 10 tokens. Instead, these players were provided with information about what Player B *would have done*, had they sent 5 or 10 tokens. However, because this treatment was hypothetical, generalization effects may be weaker than among those who gave 5 or 10 tokens, and a test of heterogeneous treatment effects can therefore be appropriate.

We find that among participants paired with a Latino partner in the first round, the difference in partner choice for the second round is driven largely by those who initially gave 5 tokens. Among this subgroup, an untrustworthy first-round interaction significantly reduced the probability of choosing Latino partners in the second round from 42.1% to 23.4% (p = .031). In contrast, among those who initially gave 0 or 10 tokens, the first-round partner’s behavior did not significantly affect partner choice in the subsequent round (p = .932 and p = .594 for 0 and 10 tokens, respectively). Among those paired with a White partner in the first round, for which the overall effect of a trustworthy interaction on selection is non-significant, none of the subgroups reached statistical significance either.

In summary, for those paired with a Latino partner in the first round, the first-round partner’s behavior significantly affected partner choice for the second round. For those paired with a White partner in the first round, however, the first-round partner’s behavior did not affect partner choice for the second round.

### The effect of a trustworthy or untrustworthy interaction on contributions

In addition to the impact on partner choice, we hypothesized that the tendency to generalize from an initial interaction to a subsequent interaction with a different person would be visible in the number of tokens sent by participants in the second round. Specifically, we hypothesized that participants paired with a trustworthy partner in the first round would send more to their partner in the second round than would participants paired with an untrustworthy partner in the first round (H3). OLS regression results indicate that an untrustworthy interaction lowers contribution in the second round by 0.093 tokens relative to those who have a trustworthy interaction (Table 2, Model 3). However, this estimate is not statistically significant (p = .170). Supplementary analyses of covariates accounting for demographic traits of respondents indicate that male respondents contributed, on average, 0.638 more tokens than female respondents (p = 0.011, model 3 in Table 2), indicating greater generosity among male respondents towards same-gender partners; however, respondent gender did not moderate the treatment effect.

**Table 2:** Regression estimates: Round 2 contributions as a function of partner ethnicity and trustworthiness in R1.

	Hypothesis tested			
Predictor	Model 3	Model 4	Model 5a	Model 5b
Untrustworthy behavior	−0.059 (0.235)	0.032 (0.325)	−0.401 (0.346)	0.135 (0.336)
Same ethnicity in both rounds		0.239 (0.339)		
Latino partner (R1)			−0.441 (0.341)	
Latino partner (R2)				−0.022 (0.336)
Untrustworthy x same ethnicity		−0.183 (0.476)		
Untrustworthy x Latino partner (R1)			0.621 (0.471)	
Untrustworthy x Latino partner (R2)				−0.378 (0.466)
N	606	606	606	606

*Note:* ‘p < 0.1; *p < 0.05; **p < 0.01; ***p < 0.001

We also expected that the effect of first-round partner’s behavior on second-round contributions would be stronger among those paired with partners of the same ethnicity in both rounds (H4). However, we did not find evidence in support of this hypothesis. In Model 4, which included an interaction term between first-round partner behavior and partner ethnic match across both rounds, the coefficient for an untrustworthy interaction was negative but insignificant (−0.003, p = 0.993), as was the coefficient for ethnic match (−0.247, p = .469). The interaction between the two terms was also not significant (p = .708).

Even when we look at the ethnicity of the partner in round 1 and round 2 separately, we again found that neither the ethnicity of the partner in round 1 (Model 5a: coefficient = −0.423, p = .220), nor in round 2 (Model 5b: coefficient = −0.02, p = .961) had an impact on contributions in round 2. The interaction terms between the first-round partner’s behavior and the ethnicity of the partner in round 1 or 2 were also found to be non-significant.

In summary, at the aggregate level, the ethnicity of partners across the two rounds had no effect on how much participants gave in the second round. The coefficients were in the anticipated direction; an untrustworthy interaction was associated with lower contributions in the subsequent round. However, this effect did not reach statistical significance in any of the models.

### Contributions—conditional average treatment effect

Here, we explore the possibility that the aggregate effects mask heterogeneity across subgroups, as we did in the analysis of partner selection. The following results are based on post-hoc analyses that were not pre-registered.

**Fig 4:**
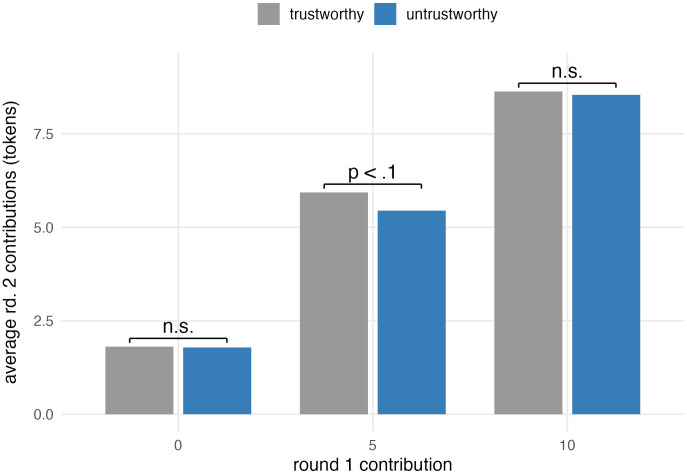
Average round 2 contribution by trust condition.

Analyses of second-round contributions among participants who did not select their partner, broken down by initial contributions (0, 5, or 10 tokens) and first-round partner behavior, did not reveal heterogeneous effects, in line with the aggregate result. On the other hand, the analyses of second-round contributions among all participants (including those who selected their partner), broken down by initial contributions and first-round partner behavior, reveal heterogeneous effects. Fig 4 shows mean second-round contributions by how much participants gave in the first round and whether the first-round partner was trustworthy or untrustworthy. Across all three subgroups, participants who had an untrustworthy partner in round 1 contributed less in round 2 compared to those who had a trustworthy partner. The decrease is most pronounced among those who initially gave 5 tokens. T-tests looking at the difference in round 2 contributions based on the first-round partner’s behavior separately by initial contribution levels (0, 5 and 10 tokens) indicate that there is no statistically significant effect of first-round partner behavior among those who initially gave 0 or 10 tokens (p = .957 and .717, respectively) (S4 Table). However, there is an effect of first-round partner behavior (d = 0.16, p = .058) among those who initially gave 5 tokens.

In summary, we do not find strong evidence to support the hypothesis that second-round contributions are affected by first-round partner behavior, except among those participants who gave 5 tokens initially. This subgroup’s responsiveness to treatment mirrors the stronger effect observed among this subgroup in the partner selection analysis. Nevertheless, given that this result is based on post-hoc analysis, follow-up studies are needed before we can confidently assert the validity of this finding.

## Discussion

Our results confirm the existence of primary transfer effects in intergroup contact, even after a single interaction with an anonymous partner online. These results complement studies that find generalization after repeated contact online [12], or in intimate, long-term encounters [17–19]. The findings stand in contrast to studies which find that the effects of contact are limited to the person with whom participants interact directly, rather than strangers from the same group [22,23]. We await research that examines the durability of the observed effects. In our experiment, the first and second rounds of the trust game were separated by just a few minutes. Moreover, given that our study was based on a non-representative sample drawn from MTurk, future work should examine whether similar patterns emerge in more diverse or probability-based samples.

Importantly, we also find that the tendency to generalize to strangers from the same group is limited to interactions with outgroup members, not ingroup members. The differential tendency to generalize from interactions with outgroup versus ingroup members can have important consequences. For one, it suggests that the actions of minority group members may be viewed as more representative of their ethnic group than those of majority group members [31,32]. In turn, minority group members may find that their actions have disproportionate consequences for others in their group, and that they themselves may be affected to a greater degree by the actions of their coethnics.

The practical implications of differential generalization may depend on a number of factors, including whether there is an asymmetry in how positive and negative behaviors are generalized. If people were to generalize more strongly from positive interactions, it would be beneficial for improving intergroup relations. Our study did not include a baseline condition against which the magnitude of the effects of trustworthy versus untrustworthy encounters could be compared. However, existing research suggests that the opposite is more likely: studies have found that outgroup members who do not conform to negative stereotypes are more often viewed as exceptions and categorized as a distinct group or subsumed into other groups [54,55], suggesting that positive new information about outgroup members is more likely to be dismissed, while negatively valenced contact tends to be more effective at highlighting boundaries and, therefore, tends to have a stronger impact than positive contact [56–58].

Our findings also draw attention to the importance of selection, which seems to be more sensitive to new information than is behavior toward outgroup members when participants *have no choice but to interact with outgroup members*. These results echo recent findings that highlight the role of selection in preventing intergroup contact from materializing [34]. Together with the positive-negative asymmetry, it implies that improvements in intergroup relations can be challenging to achieve organically, if people disproportionately generalize negative experiences with specific individuals and avoid contact with the outgroup broadly. The results imply the effects of diversification may be mixed. Diverse communities provide more opportunities for contact with trustworthy non-coethnics. However, if the balance of negative to positive encounters is too high––or, more likely, if negative encounters are made especially salient by media coverage or inflammatory political rhetoric––the weaker effects of positive encounters may not neutralize those of negative ones. The relative effects of positive and negative contact, in short, probably depend on the broader climate in which intergroup encounters unfold, an issue to which we return.

Heterogeneity in the stated effects warrants further investigation. Both in terms of selection and in contributions, participants who gave 5 tokens in the first round––those who presumably were more uncertain about their partner’s behavior––showed stronger changes in behavior in response to first-round partner behavior. On the one hand, heterogeneous effects may be an artifact of the study design. Participants who gave 0 tokens initially could give more in the second round, but not less; they could decide to be more trusting after a trustworthy first-round interaction, but they could not be less trusting after an untrustworthy interaction (they had already sent the lowest amount possible). Analogously, those who gave 10 tokens in the first round could only give less, not more. By contrast, participants who gave 5 tokens could send more in response to a trustworthy first-round interaction, or less in response to an untrustworthy one. Our subgroup analyses may reflect this feature of the study design.

On the other hand, the observed heterogeneity may reflect underlying baseline orientations towards uncertainty and its interaction with the treatment. Although subgroup differences were not theorized in the initial design of the study, the amount trustors sent in the first round may be mapped to baseline orientations towards dealing with uncertainty. When viewed from the perspective of uncertainty, participants who initially give 0 and 10 tokens can be seen as subgroups with clearer baseline expectations; those who give 0 are certain that the partner will not return any money, while those who give 10 tokens are certain that the partner will return some. Participants who give 5 tokens may be less certain, as reflected in their decision instead to “hedge their bets.” To the extent that the decision to send 5 tokens reflects uncertainty about how others will behave, this may explain why these participants react most when they acquire new information about how people behave in trust games. Future research should explore the role of uncertainty in moderating the effects of intergroup contact.

Are White participants less likely to select Latino partners and more likely to generalize from negative interactions with Latino strangers because Latinos are, on average, lower-status or because they are numerically fewer? Research in social psychology suggests that ingroup bias is consistently associated with status, independent of numerical size [59]; further, large and growing groups (as opposed to small ones) fuel concerns about intergroup status differences that exacerbate bias [60]. Still, the current design cannot disentangle the effects of status and numeric size. Nor can our experiment answer whether and how the observed effects generalize toward other racial/ethnic outgroups, including Black and Asian Americans. We would expect more similarities than differences, given that Black and Asian Americans are, like Latino Americans, both lower in status and numerically smaller than Whites. We nevertheless await research that answers these questions directly.

The study was fielded in mid-2022. Between 2021 and 2024, the United States experienced record-high immigration across its southern border [61]. And although Donald Trump was not in office in 2022, his anti-immigrant rhetoric since the 2016 election campaign legitimized the open expression of anti-immigrant and anti-Latino sentiments [62]. In our experiment, White participants might have behaved more equitably toward Latino strangers in a different period characterized by less virulent rhetoric or less undocumented immigration. However, Trump is in office once again, using inflammatory language and pursuing the mass deportation of undocumented immigrants (most of them Latino). Far from an aberration, the current climate of open hostility toward immigrants and Latinos may be a new normal.

## Supporting information

S1 Table Demographic characteristics of participants. (DOCX)

S2 Text Face recognition task – procedure and formula. (DOCX)

S3 Table Partner selection by partner ethnicity and trustworthiness in R1. (DOCX)

S4 Table Mean contributions in R2 by contribution level in R1 (full sample). (DOCX)
