# Supplementary material for: Trust over repeated interactions: Majority group members generalize more from interactions with non-coethnic partners
Source: PLoS One. 2026 Mar 10;21(3):e0341143. doi: 10.1371/journal.pone.0341143 (PMC12974844; doi:10.1371/journal.pone.0341143)
Supplement: S1 Table — (DOCX) [file pone.0341143.s001.docx]

**S1 Table. Demographic characteristics of participants**

| **Variable** | **Stats / Values** | **Total Sample (N=1255)** | | **Version 1 (N=625)** | | **Version 2 (N=630)** | |
| --- | --- | --- | --- | --- | --- | --- | --- |
|  |  | Count | % | Count | % | Count | % |
| gender | 1. Female | 469 | 37.4% | 238 | 38.1% | 231 | 36.7% |
|  | 2. Male | 785 | 62.5% | 386 | 61.8% | 399 | 63.3% |
|  | 3. Something else | 1 | 0.1% | 1 | 0.2% | 0 | 0.0% |
| income | 1. less than $10,000 | 30 | 2.4% | 17 | 2.7% | 13 | 2.1% |
|  | 2. $10,000-$19,999 | 48 | 3.8% | 28 | 4.5% | 20 | 3.2% |
|  | 3. $20,000-$29,999 | 97 | 7.7% | 51 | 8.2% | 46 | 7.3% |
|  | 4. $30,000-$39,999 | 93 | 7.4% | 41 | 6.6% | 52 | 8.3% |
|  | 5. $40,000-$49,999 | 199 | 15.9% | 97 | 15.5% | 102 | 16.2% |
|  | 6. $50,000-$59,999 | 253 | 20.2% | 130 | 20.8% | 123 | 19.5% |
|  | 7. $60,000-$69,999 | 102 | 8.1% | 55 | 8.8% | 47 | 7.5% |
|  | 8. $70,000-$79,999 | 197 | 15.7% | 100 | 16.0% | 97 | 15.4% |
|  | 9. $80,000-$89,999 | 76 | 6.1% | 27 | 4.3% | 49 | 7.8% |
|  | 10. $90,000-$99,999 | 84 | 6.7% | 36 | 5.8% | 48 | 7.6% |
|  | 11. $100,000-$149,999 | 62 | 4.9% | 32 | 5.1% | 30 | 4.8% |
|  | 12. $150,000 or more | 14 | 1.1% | 11 | 1.8% | 3 | 0.5% |
| party affiliation | 1. Democrat | 785 | 62.5% | 386 | 61.8% | 399 | 63.3% |
|  | 2. Independent | 142 | 11.3% | 77 | 12.3% | 65 | 10.3% |
|  | 3. Republican | 324 | 25.8% | 161 | 25.8% | 163 | 25.9% |
|  | 4. Something else | 4 | 0.3% | 1 | 0.2% | 3 | 0.5% |
| age | 1. 18-24 years | 68 | 5.4% | 28 | 4.5% | 40 | 6.3% |
|  | 2. 25-29 years | 336 | 26.8% | 169 | 27.0% | 167 | 26.5% |
|  | 3. 30-34 years | 288 | 22.9% | 130 | 20.8% | 158 | 25.1% |
|  | 4. 35-39 years | 228 | 18.2% | 121 | 19.4% | 107 | 17.0% |
|  | 5. 40-44 years | 119 | 9.5% | 60 | 9.6% | 59 | 9.4% |
|  | 6. 45-49 years | 69 | 5.5% | 33 | 5.3% | 36 | 5.7% |
|  | 7. 50-54 years | 50 | 4.0% | 31 | 5.0% | 19 | 3.0% |
|  | 8. 55-59 years | 53 | 4.2% | 27 | 4.3% | 26 | 4.1% |
|  | 9. 60-64 years | 29 | 2.3% | 17 | 2.7% | 12 | 1.9% |
|  | 10. 65 years or older | 15 | 1.2% | 9 | 1.4% | 6 | 1.0% |
