# Supplementary material for: Trust over repeated interactions: Majority group members generalize more from interactions with non-coethnic partners
Source: PLoS One. 2026 Mar 10;21(3):e0341143. doi: 10.1371/journal.pone.0341143 (PMC12974844; doi:10.1371/journal.pone.0341143)
Supplement: S2 Text — (DOCX) [file pone.0341143.s002.docx]

**S2 Text. Face Recognition Task – Procedure and Formula**
