# Supplementary material for: Trust over repeated interactions: Majority group members generalize more from interactions with non-coethnic partners
Source: PLoS One. 2026 Mar 10;21(3):e0341143. doi: 10.1371/journal.pone.0341143 (PMC12974844; doi:10.1371/journal.pone.0341143)
Supplement: S3 Table — (DOCX) [file pone.0341143.s003.docx]

**S3 Table. Partner selection by partner ethnicity and trustworthiness in R1**

|  | Round 1 partner: **White** | | Round 1 partner: **Latino** | |
| --- | --- | --- | --- | --- |
|  | Round 2 partner choice | | Round 2 partner choice | |
| interaction | White | Latino | White | Latino |
| trustworthy | 0.625 | 0.375 | 0.534 | 0.466 |
| untrustworthy | 0.573 | 0.427 | 0.639 | 0.361 |
|  | χ²=0.67, df=1, N= 327, p = 0.412 | | χ²=2.88, df=1, N= 303, p = 0.090 | |
