# Supplementary material for: Trust over repeated interactions: Majority group members generalize more from interactions with non-coethnic partners
Source: PLoS One. 2026 Mar 10;21(3):e0341143. doi: 10.1371/journal.pone.0341143 (PMC12974844; doi:10.1371/journal.pone.0341143)
Supplement: S4 Table — (DOCX) [file pone.0341143.s004.docx]

**S4 Table. Mean contributions in R2 by contribution level in R1 (full sample)**

|  | average round 2 contributions | |  |  |
| --- | --- | --- | --- | --- |
| Initial contribution | trustworthy (tokens) | untrustworthy (tokens) | t(df) | *p* |
| 0 tokens | 1.809 | 1.786 | -0.05(189.92) | 0.957 |
| 5 tokens | 5.933 | 5.445 | -1.90(539.08) | 0.058’ |
| 10 tokens | 8.633 | 8.541 | -0.36(471.93) | 0.717 |
| *Note:* ‘p<0.1; *p<0.05; **p<0.01; ***p<0.001 | | | | |
